# Fibroblast Growth Factor 22 Is Not Essential for Skin Development and Repair but Plays a Role in Tumorigenesis

**DOI:** 10.1371/journal.pone.0039436

**Published:** 2012-06-21

**Authors:** Monika Jarosz, Luisa Robbez-Masson, Athina-Myrto Chioni, Barbara Cross, Ian Rosewell, Richard Grose

**Affiliations:** 1 Centre for Tumour Biology, Barts Cancer Institute – a Cancer Research UK Centre of Excellence, Queen Mary University of London, London, United Kingdom; 2 Biological Services, Clare Hall Laboratories, Cancer Research UK London Research Institute, South Mimms, Herts, United Kingdom; The University of Hong Kong, Hong Kong

## Abstract

Fibroblast Growth Factors play critical roles during development, tissue homeostasis and repair by controlling cell proliferation, survival, migration and differentiation. Of the 22 mammalian FGFs, FGF22, a member of the FGF7/10/22 subfamily, has been shown to have a clear role in synaptogenesis, but its roles in other tissues have not been studied. We have investigated the *in vivo* functions of FGF22 in mice. *Fgf22* null animals were viable, fertile and did not display any obvious abnormalities. Despite the known expression profile of FGF22 in the skin, no differences in either skin or pelage were observed, demonstrating that FGF22 is dispensable during embryogenesis and in unchallenged adult skin. Mice lacking FGF22 were able to heal acute wounds just as efficiently as wild type mice. However, classical two-step skin carcinogenesis challenge revealed that FGF22 null mice developed fewer papillomas than wild type controls, suggesting a potential pro-oncogenic role for FGF22 in the skin.

## Introduction

Fibroblast Growth Factors (FGFs) are a large family of 22 signalling molecules, responsible for regulating a range of cellular processes including proliferation, survival, migration, differentiation and response to injury [1]. Their various roles have been delineated through a large number of genetically modified mouse studies (reviewed in [2]). They act, for the most part, as secreted growth factors, which bind to receptor tyrosine kinases on nearby cells. FGFs can be grouped into subfamilies, based on sequence similarity and receptor specificity [1], [3], [4].

The biological activities of FGFs are mediated by high affinity cell surface tyrosine kinase receptors. FGF7/10/22 subfamily members activate two main receptors: FGFR1b and FGFR2b, although they signal preferentially, and exclusively in the case of FGF7, via FGFR2b [4], [5]. Striking phenotypic similarities between *fgf10* and *fgfr2b* knockout mice [6], [7], [8], together with rather modest phenotypes of *fgf7* [9] and *fgfr1b* [10] knockout mice, have established the FGF10-FGFR2b axis as a key ligand-receptor partnership in embryogenesis. FGF22 is a relatively understudied member of the FGF7/10/22 subfamily [11]. In the skin, FGFs 7, 10 and 22 act predominately on cells of epithelial origin but, uniquely, FGF22 is expressed by epithelial cells [12], suggesting a cell autonomous role.

Expression of FGF22 is relatively restricted. It first was detected, by Northern blot, in placenta, brain and skin [11]. In murine skin, expression of FGF22 begins at embryonic day 16.5, as determined by RNase protection assay [12]. In adult mouse skin, FGF22 is expressed in the inner root sheath (IRS) of the hair follicle [11] and in the interfollicular epidermis [12]. During hair development, the dermal papilla, a cluster of specialised mesenchymal cells within the dermis, signals to epidermal follicular stem cells to proliferate and differentiate into IRS, medulla and cortex cells, which together with the cuticle cells undergo terminal differentiation to form the mature hair fibre [13]. FGF7 and FGF10 are expressed in the dermal papilla of the hair follicle [14] and *fgf7* knockout mice displayed a mild hair phenotype, with male mice developing greasy, matted hair with age [9]. Furthermore, transgenic mice overexpressing FGF7 in the epidermis demonstrated abnormal patterns of hair growth [15], and subcutaneous or intraperitoneal injections of recombinant FGF7 into nude mice stimulated hair growth by extending the anagen phase of the hair cycle [16]. Since both *fgf10* and *fgfr2b* knockout mice die at birth, their hair phenotype is hard to study. Nevertheless, late stage *fgfr2b* knockout embryos showed a reduction in hair follicle development, with significantly fewer, developmentally retarded, hair follicles relative to wild type littermates [17]. Skin grafting studies, using late stage *fgfr2b* null and wild type foetuses, showed that FGFR2b signalling was crucial for normal epidermal growth and development as well as for subsequent hair follicle morphogenesis [18]. Transgenic mice expressing dominant-negative FGFR2b in differentiating hair keratinocytes developed abnormally thin, but otherwise normal, hairs characterised by single columns of medulla cells in all hair types [13]. Mice lacking *fgfr2b* only in the epidermis developed similarly thin and silky pelage hair [19].

FGFs 7, 10 and 22 show distinct temporal expression patterns through the murine hair cycle, with both FGF7 and FGF10 expressed highly at anagen V (day 8), when hair grows vigorously, and FGF22 expression strong at anagen VI (day 18), when hair follicle reaches its maximum length [20]. This pattern of expression recapitulates that seen during the wound healing process [12].

FGF7 is expressed weakly in normal murine and human skin, but, upon injury, its expression is up-regulated dramatically [21]. FGF10 levels also increase rapidly following wounding [22] and levels of both growth factors decline once re-epithelialisation is complete [20]. In contrast, FGF22 expression declines during the first days after wounding and remains low until day 5 after injury. Subsequently, the expression increases above basal levels at day 7 after wounding and remains elevated until day 13, being localised to the hyperthickened epidermis of fully healed wounds [12]. *Fgf7* knockout mice showed no defect in their ability to repair incisional wounds and the proliferation rate of keratinocytes at the wound edge was not impaired [9]. This was unexpected, since transgenic animals expressing a kinase-deficient, dominant-negative, FGFR2b displayed a severe delay in wound re-epithelialisation, with an 80–90% reduction in the number of proliferating keratinocytes in the hyperproliferative epithelium of five day old excisional wounds, compared with control mice [23]. Truncated FGFR2b abrogates the effects of FGF7, FGF10, FGF1 and FGF3, thus blocking the potential ligand redundancy seen in *fgf7* knockout mice, where FGF10 may be sufficient to drive normal repair. Supporting this hypothesis, a significant delay in wound re-epithelialisation was seen in mice lacking dendritic epidermal T cells (DETC), an important source of FGF7 and FGF10 in the healing wound [24]. Finally, mice lacking FGFR1b, a receptor for FGF10 but not FGF7, did not display abnormalities in skin development or repair [10].

Epidermal specific deletion of *fgfr2b* resulted in a loss of sebaceous glands and abnormal hair development, with mice developing thickened epidermis over time and showing exquisite sensitivity to chemical-induced skin carcinogenesis [19]. Deletion of both *fgfr1* and *fgfr2* led to an even more extreme phenotype, with dramatic hair loss and epidermal hyperthickening, demonstrating the importance of FGFR signaling in skin homeostasis [25].

What emerges from the above picture is that there is a clear role for FGF signalling in the wound healing process and that this role is protected by redundancy at the level of both ligand and receptor. Our current study builds on these data as the first to investigate the role of FGF22 in the skin, using a germline knockout approach.

## Results

### Generation of *fgf22* Knockout Mice

**Figure 1:**
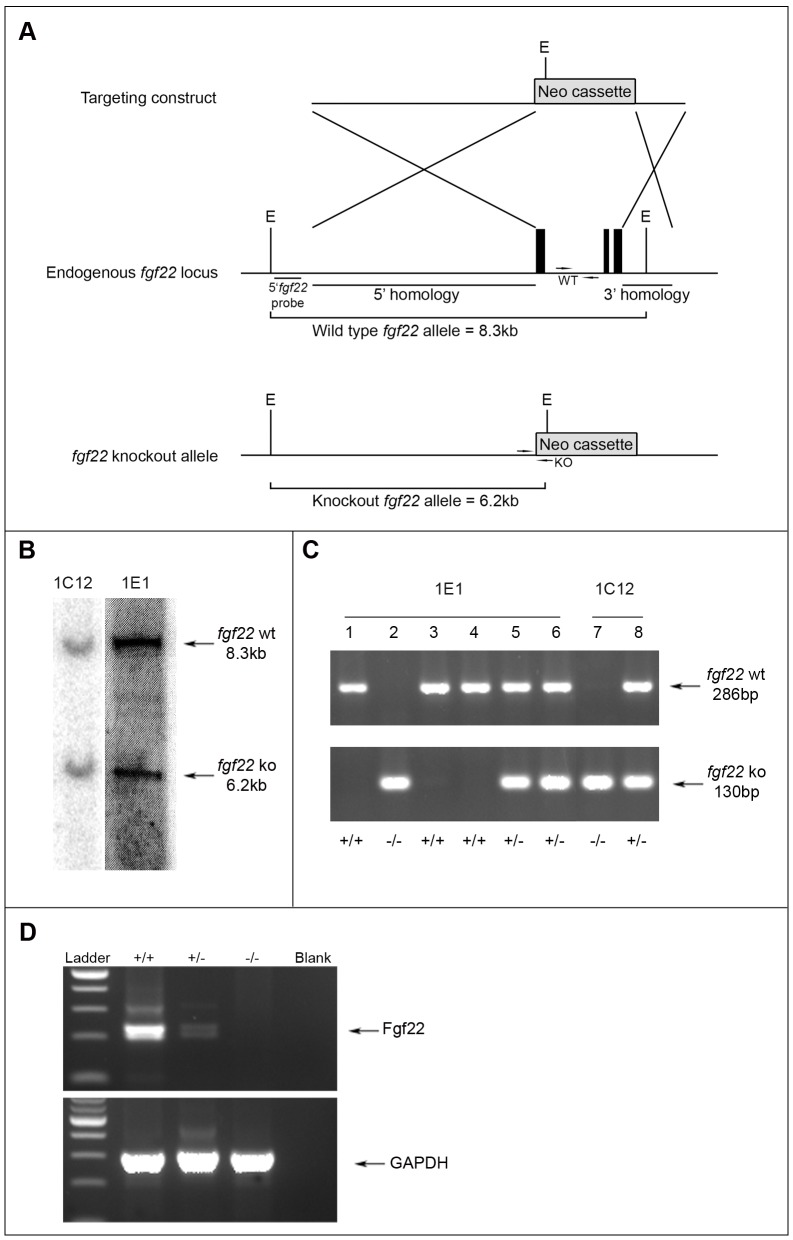
Generation of *fgf22* knockout mice. A) Targeting strategy for the *fgf22* knockout mouse. Schematic representations of the endogenous mouse *fgf22* locus (middle), targeting construct (top) and disrupted allele (bottom). Black boxes in the wild type allele represent exons 1 to 3. E indicates *EcoRI* restriction enzyme recognition sites. PCR primers used for genotyping are represented by arrows. B) Southern blots showing successful recombination in ES cells used for generation of *fgf22* null mice. Of 192 ES clones analysed, two scored positive for homologous recombination (1C12 and 1E1) and displayed identical hybridisation patterns. C) PCR analysis of genomic DNA isolated from ear snips of an heterozygous breeding pair of both identified mutant clones. *Fgf22* wt (+/+) samples show a single band at 286 bp (mice 1, 3 and 4), *fgf22* ko (−/−) display a single ko allele at 130 bp (mice 2 and 7) and heterozygous (+/−) samples amplify both wt and ko alleles (mice 5, 6 and 8). D) Confirmation of gene deletion. RT-PCR analysis of cDNA generated from mouse brain. Samples from wild type mice (+/+) display an intense *fgf22* band, *fgf22* heterozygous samples (+/−) display same size band of decreased intensity and *fgf22* knockout samples (−/−) lack the presence of a correct size band. GAPDH primers were used as a control for RNA quality and concentration. Blank represents PCR reaction mix without cDNA.

Mice lacking *fgf22* were generated by replacing the *fgf22* genomic sequence with a Neomycin selection cassette, such that homologous recombination eliminated the entire coding region of the *fgf22* gene (exons 1, 2 and 3) (Fig. 1A). Two G418-resistant clones were identified as homologous recombinants by Southern blot analysis, after EcoRI digestion (Fig. 1B), using a probe external to the homologous sequence to confirm correct targeting. The two mutant clones were injected separately into C57Bl6/J blastocysts. Male chimaeric animals of at least 70% 129ola contribution (as judged by coat colour) were mated with C57Bl6/J females and the offspring of these mice were screened for germline transmission. Heterozygous offspring were mated to produce homozygous mutants, which were born at the expected Mendelian frequency of 25% for both clones (1C12 and 1E1). PCR genotyping was performed to identify *fgf22* knockout mice, with primers for the wild type (wt) allele generating a 286 bp product and null (ko) allele a 130 bp product (Fig. 1C). Knockout mice from both clones were indistinguishable in terms of cutaneous histology (data not shown), so just one clone (1E1) was used for the functional investigations presented.

To verify that targeted homologous recombination resulted in abrogation of FGF22 mRNA expression, total RNA was isolated from mouse brain, known to express FGF22 at a detectable level [11]. Following cDNA synthesis, PCR was performed using primers recognising exon1 and exon3 of the *fgf22* coding sequence. As expected, relative to control wild type mouse brain, FGF22 mRNA expression was reduced in heterozygous brains and completely absent in knockout mice (Fig. 1D). Unfortunately, we were unable to detect endogenous levels of FGF22 protein in cells or tissue from control mice, using a polyclonal antiserum known to detect FGF22 expression in cells transfected with a constitutive FGF22 expression construct [12].

### Characterisation of *fgf22* Knockout Mice

**Figure 2:**
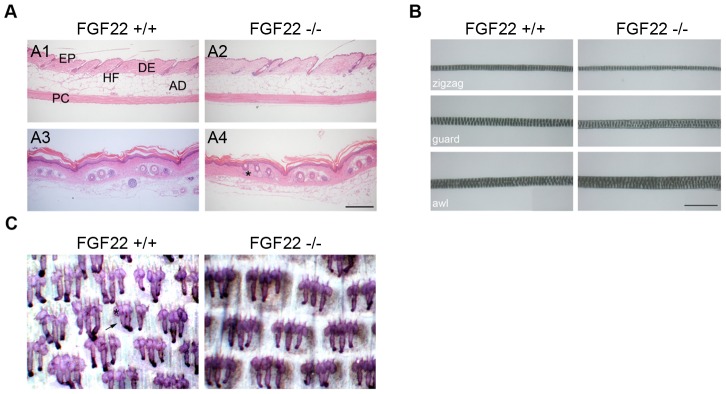
Normal skin and pelage hair in *fgf22* knockout mice. A) Histological analysis of H&E stained back (A1 and A2) and tail (A3 and A4) skin sections from four-month-old *fgf22* wild type and knockout mice. No differences were observed in skin thickness or morphology, when back skin or tail skin sections from control and *fgf22* null mice were compared. (EP epidermis, DE dermis, AD adipose tissue, PC *panniculus carnosus*, HF hair follicle, * sebaceous gland). Scale bar (200 µm). B) Comparison of pelage hair structure. All major hair shaft types (zigzag, guard and awl/auchene) were present in both wild type and knockout animals. The morphology of different hair types showed no difference between wild type and *fgf22* knockout mice as shown by light microscopic analysis. Hairs were plucked from eight-week-old wild type mice (n = 100 hairs) and compared with hairs of *fgf22* knockout mice (n = 100 hairs). Scale bar  = 200 µm. C) Tail epidermis whole-mount preparations from 10 weeks old mice, stained with Mayer’s haemalum, revealed no difference between wild type and *fgf22* knockout mice in terms of sebaceous gland number and morphology. Hair follicle is indicated by arrow and sebaceous gland by asterisk.

*Fgf22* knockout animals were viable, fertile and did not display any obvious abnormalities, demonstrating that FGF22 is not essential for embryonic development. As FGF22 is both a ligand for FGFR2b and is expressed in the skin, we first performed a detailed analysis of those epidermal structures affected by lack of FGFR2b in the epidermis. H&E stained sections of back and tail skin from wild type and knockout mice also showed identical histologically, in terms of epidermal, dermal and adipose thickness as well as hair follicle frequency (Fig. 2A1–A4). There were no evident differences in pelage growth or sebaceous gland morphology between knockout and wild type mice. All the major hair types (zigzag, guard and awl/auchene) were present in knockout mouse back skin, at the expected frequencies (70% zigzag, 28% awl/auchene and 2% guard hairs) and these hairs did not demonstrate any morphological differences when compared with those of wild type littermates (Fig. 2B) or compared to published mouse hair phenotypes [26]. Furthermore, average length of zigzag hairs (8.7 mm) was identical between genotypes. Whole-mount staining of the sebaceous glands in adult tail skin confirmed no differences between knockout and wild type mice (Fig. 2C).

**Figure 3:**
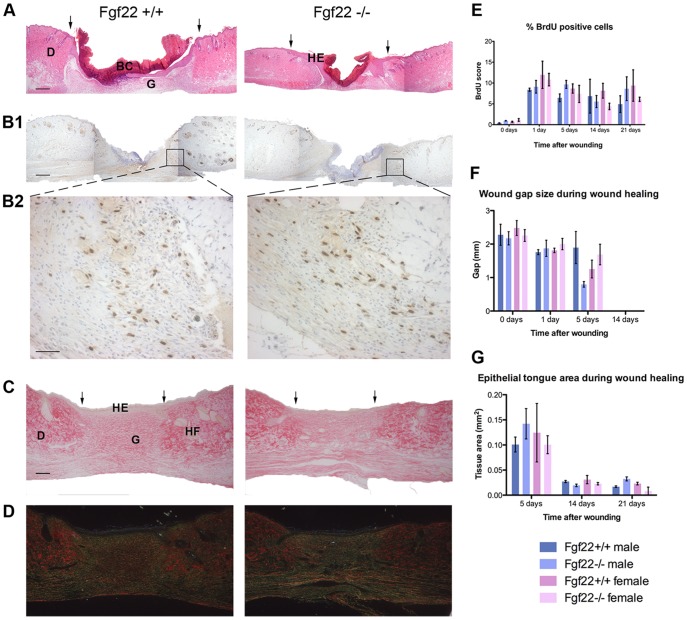
Normal wound repair in *fgf22* knockout mice. A) H&E stained tissue sections of 3 mm punch biopsy wounds 5 days after wounding. B1–B2) Tissue sections immunostained for 5-bromo-2-deoxyuridine. High power fields in B2) focus on BrdU-positive cells (brown) at the wound site. C and D) Sirius red staining of sections from wild type and *fgf22* knockout male mice skin 14 days post-wounding. C) represents the same section in bright field as D) in dark field. The spectrum of colours from green, yellow, orange and red progressively exhibits the packing of collagen molecules. (D dermis, HE hyperproliferative epidermis, HF hair follicle, G granulation tissue, BC blood clot. Wound margins are indicated by arrows). Scale bar (200 µm) in A, B1, C, D; (50 µm) in B2. E). The percentage positive BrdU score was calculated from immunostained sections from B1 by counting the total number of positive and negative cells within the wound margins and then deriving the percentage of positive cells. Plots represent mean of three independent wounds per genotype per time point where two slides per wound were counted. Error bars represent SEM. F) and G) Morphometric analysis of wound repair. Morphometric measurements of wounds were performed on three independent wounds for each genotype and each time point. F) Wound gap was calculated as the distance between two margins of the inward growing epithelium and was measured three times for each wound section. G) The area of migrating epithelial tongue was also measured. Error bars represent SEM.

Since there were no defects evident in the unchallenged skin of *fgf22* knockout mice, we investigated their capacity to respond to skin injury and carcinogenic insult, both of which have been shown to depend at least in part on FGF signalling. Wound healing studies, where mice were subject to 3 mm diameter full thickness punch biopsy wounds, showed that mice lacking FGF22 were able to heal acute wounds just as efficiently as wild type mice, although 5 days after wounding knockout male wounds appeared to have healed faster (Fig. 3A), with more efficient re-epithelialisation (Fig. 3F) and a high rate of proliferation of cells involved in the process at this time-point (Fig. 3B1 and B2, quantified in 3E). However, at all other time-points during wound healing, morphometric analysis showed very similar values for both male and female *fgf22* wild type and knockout animals (Fig. 3E, F and G). Moreover, Sirius red staining of 14 day old wounds from male *fgf22* knockout and wild type controls showed no difference in collagen fibre reorganization during healing (Fig. 3C and D).

**Figure 4:**
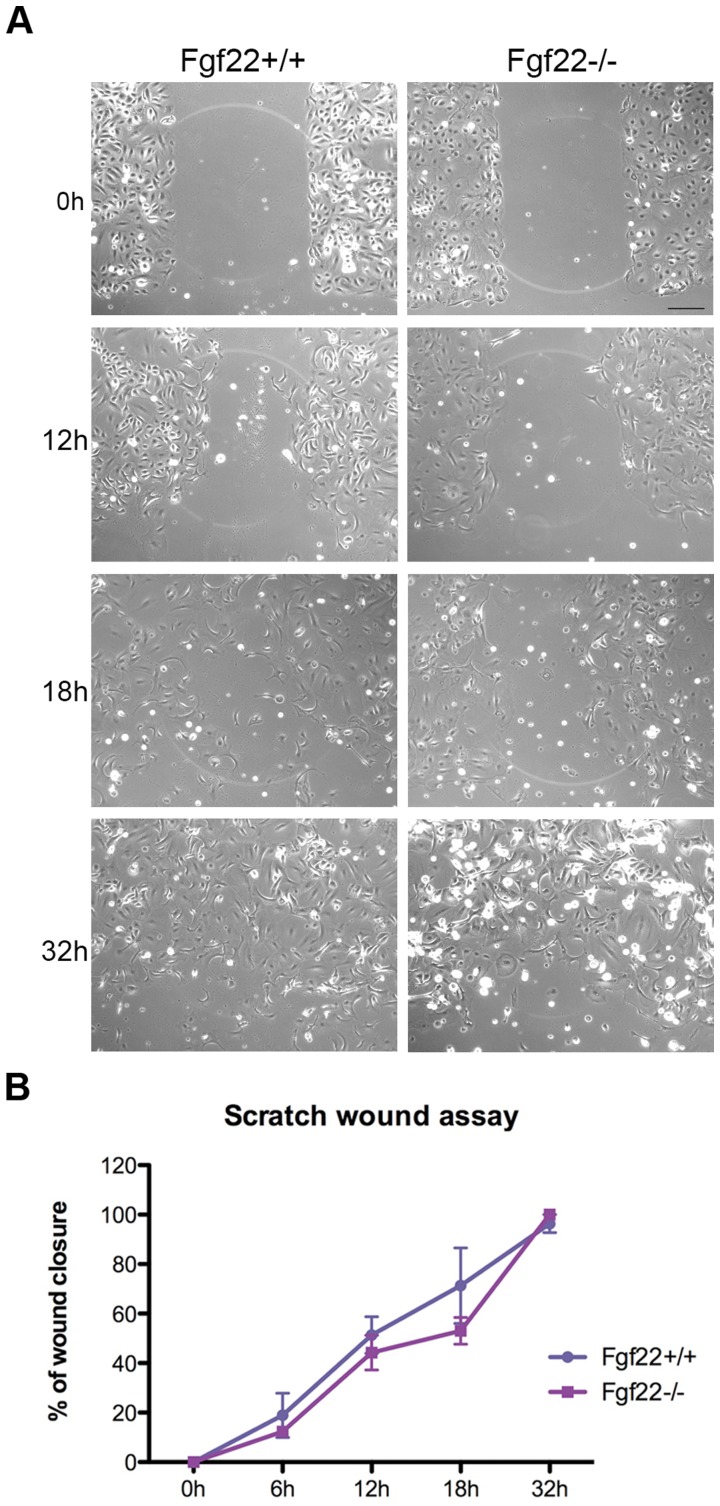
*In vitro* wound healing. Scratch wound assay was performed on primary keratinocytes isolated from wild type and *fgf22* knockout male back skin. A) Photographs show the migration of wild type and knockout primary keratinocytes to wounded regions of the monolayer culture at 0, 12, 18 and 32 h post wounding. Scale bar (200 µm). B) Wound closure was monitored, photographed and measured at all timepoints after wounding. Error bars represent SEM.

To complement the *in vivo* wound healing study, we isolated keratinocytes from back skin of wild type and knockout mice and compared their behaviour in a 2D scratch wound assay. Keratinocytes from *fgf22* knockout mice were able to close the wound gap just as effectively and rapidly as those from wild type animals at all timepoints (Fig. 4A and B).

**Figure 5:**
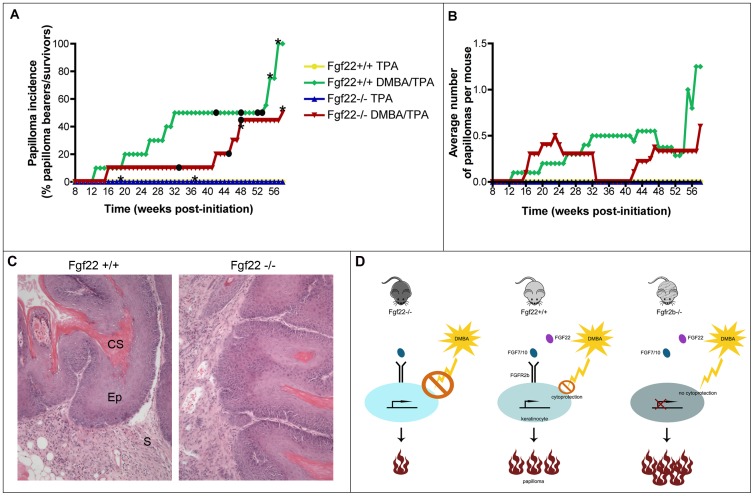
DMBA-induced tumorigenesis study. Cohorts of female mice (n = 10) were subjected to classical two-step skin carcinogenesis treatment. A) At 58 weeks post initiation, when the experiment was terminated, 100% of DMBA treated wild type mice had developed papillomas while only 50% of knockout treated mice had papillomas. Control TPA treated mice of both genotypes never developed any neoplastic skin lesions. B) Average number of papillomas per mouse at the indicated times after the start of DMBA/TPA treatment. With the exception of one outlier knockout female that developed five papillomas, the remaining knockout mice never developed more than one papilloma per mouse. The maximum number of papillomas on a DMBA treated wild type female was two. In addition to regression of some papillomas over time, when mice died, the average papilloma count was re-calculated for the remaining live mice, thus the average count could go up and down over the course of the experiment. Circles represent mice killed due to papillomas reaching the maximum size specified by Home Office licence. Asterisks represent deaths due to unrelated reasons. C) H&E staining of 4 µm sections from papillomas of wild type and *fgf22* knockout mice showed identical histology, with exophytic growth and no sign of epithelial invasion (CS cornified squames; Ep epithelium; S stroma). D) Cartoon illustrating difference in response of wild type, *fgf22* knockout and skin-specific *fgfr2b* knockout mice to chemically induced carcinogenesis and a proposed model for the phenotypes seen. We hypothesise that lack of downstream cytoprotective signalling mediated via Fgfr2b could potentially promote a more pro-tumorigenic phenotype.

Finally, we challenged the mice with a classical two-step skin carcinogenesis assay using DMBA and TPA. These experiments were performed on female mice, since they involved long-term observation and multiple mice could be kept together. When cohorts of *fgf22* knockout female mice were subjected to two-step (DMBA/TPA) skin carcinogenesis treatment, they developed significantly less papillomas than wild type mice. *Fgf22* wild type and knockout mice subjected to TPA treatment alone never developed papillomas (Fig. 5A). Over the duration of the experiment, only 50% of DMBA treated knockout mice developed papillomas, compared to 100% of treated wild type mice (Fig. 5A). The time when 50% of mice developed papillomas was delayed by 16 weeks in *fgf22* knockout mice compared with wild type controls (Fig. 5A). Tumour multiplicity was difficult to judge since these studies were performed on a tumour refractory C57Bl6/J background, so few papillomas formed during the study. With the exception of one outlier knockout female that developed five papillomas, the remaining knockout mice never developed more than one papilloma per mouse. The maximum number of papillomas on a DMBA treated wild type female was two (Fig. 5B). Papillomas that did develop showed identical histology between genotypes, with an exophytic appearance and no areas of epithelial invasion (Fig. 5C).

**Figure 6:**
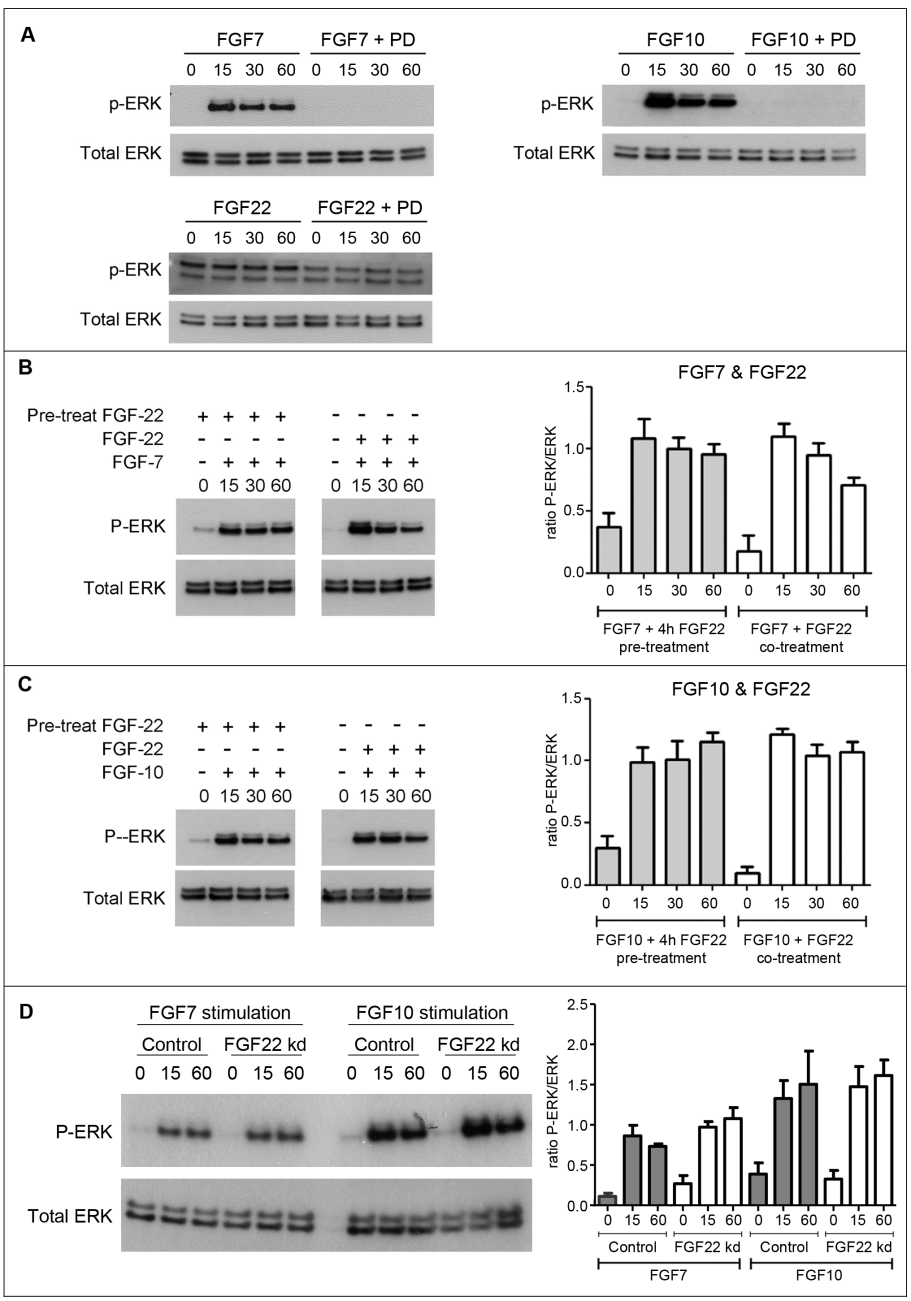
FGF signaling in HaCaT cells. A) HaCaT keratinocytes that had been serum-starved overnight were stimulated with FGF7, FGF10 or FGF22 (100 ng/ml) for 0, 15, 30 or 60 minutes and lysed in sample buffer prior to Western blotting. FGF7 and FGF10 elicited rapid ERK phosphorylation, whereas FGF22 yielded no significant response (phospho-ERK staining appears stronger due to increased length of exposure – to allow visualization of bands). B and C) Serum-starved HaCaT cells were stimulated as above with 100 ng/ml FGF7 (B) or FGF10 (C) either following a 4 h pre-treatment with FGF22 (100 ng/ml) or concomitant with FGF22 treatment. In each case, experiments were repeated (n = 5) and bands quantified using ImageJ (bars represent SEM). No reduction in ERK signalling was observed in either case. D) Forty-eight hours post-transfection with scrambled control non-targeting siRNA or FGF22 siRNA (both at 10 nM), serum-starved HaCaT cells were stimulated with either FGF7 or FGF10 (both 100 ng/ml) for 15 or 60 minutes and levels of phospho-ERK and total ERK analysed by Western blot as described above. No difference in ERK phosphorylation was detected in either case, with experiments repeated (n = 3) and bands quantified using Image (bars represent SEM).

These findings raised the question of whether FGF22 might interfere with the tumour-suppressive effect of FGFR2b (Fig. 5D). We therefore investigated whether treatment with FGF22 altered the biochemical response to FGF treatment in HaCaT cells, an immortalised human keratinocyte cell line [27]. In order to ensure that our HaCaT cell line exhibited a standard response to FGF stimulation, serum starved cells were stimulated with FGFs 7, 10 and 22 (100 ng/ml) in the presence of Heparin (300 ng/ml) in serum free media for 15, 30 and 60 minutes. Western blotting using an antibody specific to phosphorylated ERK confirmed that stimulation with FGFs 7 and 10 triggered rapid ERK phosphorylation, and this response was blocked by pre-treatment with the FGFR inhibitor, PD173074 (1.7 µM; 30 minutes; Fig. 6A). In contrast, FGF22 elicited no such response, with P-ERK levels remaining the same as in unstimulated cells (Fig. 6A; NB: blot was exposed for longer time relative to FGF7 and FGF10 to allow visualisation of the P-ERK signal). Total ERK levels from the same blot stripped and re-probed are shown as a loading control.

To investigate whether FGF22 might modulate the capacity of FGF7 and 10 to activate ERK signalling, HaCaT cells were treated with FGF7 and 10 either concomitantly with FGF22 or following a four hour pre-treatment with FGF22 (all at 100 ng/ml; Fig. 6B and C). As above, cells were lysed after 0, 15, 30 and 60 minutes and lysates analysed by Western blot. Neither pre-treatment (four hours at 100 ng/ml) or co-treatment with FGF22 showed any effect on the timecourse of ERK phosphorylation elicited by stimulation with FGF7 or FGF10 (Fig. 6B and C respectively), although basal levels of ERK phosphorylation were raised very slightly by pre-treatment with FGF22, as shown by ImageJ quantitation of five independent experiments. Furthermore, we used an siRNA pool to knock down FGF22 expression in HaCaT cells (Sup Fig. S1) and confirmed that this also had no effect on response to stimulation with either FGF7 or 10 (Fig. 6D). Expression levels of the FGF7 target gene *NRF2* were also unaltered in knockdown cells (Sup Fig. S1). Finally, performing Realtime RT-PCR on RNA isolated from back skin of eight-week-old control and knockout female mice, we confirmed that expression levels of FGF7 and FGF10 were not altered significantly between genotypes (Sup Fig. S2).

## Discussion

Numerous studies on FGF7, FGF10 and their receptors have shown the importance of this subfamily of FGFs for normal embryonic development and adult tissue homeostasis. However, little is known about the role and importance of FGF22 in these processes. Previous studies have shown that FGF22 is expressed in the brain, where it is essential for the development of excitatory synapses [28]. Furthermore, FGF22 deficient mice, generated commercially (Deltagen, San Mateo, CA), are resistant to pentylenetetrazol-induced epileptic seizures [29], though lack of FGF22 does not affect behaviour in unchallenged mice.

FGF22 is expressed in the inner root sheath of the hair follicle [11] and has been shown to bind to FGFR2b [4]. Since there was a clear defect in hair development in *fgfr2b* null skin [19], we analysed the hair of *fgf22* knockout mice. All three major hair types were present in identical proportions to those seen in wild type littermates and no effects on sebaceous gland morphology were observed, suggesting either that redundancy prevents occurrence of an overt phenotype in the *fgf22* knockout mouse, or that its role is non-essential in unchallenged skin. Recent studies have established that signaling downstream of FGFR2b and FGFR1b is essential for skin homeostasis and epidermal barrier function, with skin specific FGFR1/2 double knockout mice having a defective skin barrier and developing epidermal hyperthickening with age, as a result of chronic inflammation [25]. Given that *fgf22* knockout mice do not phenocopy those changes, FGF22 clearly is not essential for normal skin physiology.

Therefore, we analysed the capacity of FGF22 null mouse skin to respond to a challenging stimulus; either to wounding (Fig. 3) or to classical two-step skin carcinogenesis (Fig. 5), since both of these interventions cause an increase in keratinocyte proliferation; either transiently in the case of wound repair or more permanently in the case of carcinogenesis. These studies allowed us to question whether FGF22 was capable of driving keratinocyte differentiation and/or inhibiting proliferation.

During wound healing, in contrast to FGF7 and FGF10, which appear to drive the healing process, FGF22 expression is up regulated towards the end of repair [12]. Thus, we hypothesised that it may act as a negative regulator of FGFR2 signalling, either decreasing proliferation or increasing differentiation, to safeguard against over-proliferation of keratinocytes. However, *fgf22* knockout mice did not display any defects in wound healing (Fig. 3). It is possible that, as repair progresses, the decrease in expression of pro-mitogenic ligands is sufficient to dampen the epithelial healing process. This apparent redundancy in the FGF-FGFR axis during repair is not without precedent, as mice lacking *fgf7* or *fgfr1b* showed no wound healing phenotype, despite expression of these molecules being reported during the repair process [9], [12]. Support for a redundant role of FGF22 in repair comes from the lack of difference in *in vitro* wound closure by primary keratinocytes isolated from wild type and knockout mice (Fig. 4).

Our skin carcinogenesis data suggest that FGF22 may play a role in skin carcinogenesis, with the decreased sensitivity to chemically induced carcinogenesis in *fgf22* knockout mice implying that basal expression of FGF22 confers sensitivity to skin carcinogenesis. In contrast, mice lacking FGFR2b in the skin develop spontaneous papillomas and show great sensitivity to chemically induced carcinogenesis [19]. Thus the ligand and its receptor appear to have antagonizing effects, with a tumour suppressive receptor, FGFR2b, appearing to be activated by a putative oncogenic ligand, FGF22. One model that might explain this is that FGF22 might act to antagonise the tumour suppressive role of FGFR2b, by competing with the protective ligands FGF7 and FGF10 (Fig. 5D). In this case, lack of FGF22 would cause increased signalling via FGF7 and FGF10. However, our cell-based investigations in the spontaneously immortalized HaCaT keratinocyte cell line showed FGF22 to have no effect on FGF7- or FGF10-dependent ERK activation (Fig. 6 and Sup Fig. S2).

FGF7 is known to regulate genes that encode for mediators of cytoprotection, including the antioxidant Peroxiredoxin VI and the transcription factor Nrf2 [30], [31], [32], [33]. In *fgfr2b* knockout mice, this cytoprotective mechanism is impaired due to lack of signalling receptor, and this most likely contributes to the dramatic incidence of papillomas in response to chemically induced carcinogenesis. Therefore we hypothesised that in *fgf22* knockout mice, where FGF7 and/or FGF10 do not have to compete with FGF22 for receptor binding, it may allow stronger FGF-mediated cytoprotective signalling and, consequently, reduced papilloma formation (Fig. 5D). However, knock down of endogenous FGF22 mRNA in HaCaT cells did not result in a change in expression of NRF-2, suggesting that this potential mechanism needs further investigation. Due to the inability to detect FGF22 protein by western blot, we cannot absolutely exclude that FGF22 protein levels remain unaffected despite clear mRNA knock down. FGF7, in particular, is known to be cytoprotective for keratinocytes, with a recombinant form of FGF7 used clinically in the treatment of oral mucositis in patients undergoing bone marrow transplantation [34]. Future studies generating mice over-expressing FGF22 in the skin will allow us to address this mechanism. Taken together, our data provide evidence that FGF22 is not essential for skin development or repair but may play an important role in skin tumorigenesis, though the mechanism remains to be determined.

## Materials and Methods

### Generation of *fgf22* Knockout Mice

A gene targeting construct was made by flanking a *Neo* selection marker cassette with fragments of DNA homologous to the *fgf22* genomic locus. The 5′ arm consisted of 5 kb of genomic sequence 5′ to the translation start site of *fgf22*, in exon 1, and the 3′ arm comprised 1.2 kb sequence 3′ to exon 3 of *fgf22*. 129ola mouse embryonic stem cells were used as a genomic DNA source. The targeting vector, constructed in pBluescript SK^+^ (Stratagene, La Jolla, Ca, USA), thus was designed to eliminate the entire coding sequence of *fgf22* by deleting the coding region of exon 1 and the whole of exons 2 and 3. The targeting vector DNA was transfected into isogenic 129ola ES cells and G418 resistant clones were screened by Southern blot for correct homologous recombination. Of a total of 192 ES clones analysed, 2 correctly targeted clones displayed identical hybridisation patterns and were injected separately into C57Bl6/J blastocysts. The resulting chimeras were mated with wildtype C57/BL6 female mice to obtain germline transmission.

### Southern Blotting

Genomic ES cell DNA was digested overnight with EcoRI and separated by electrophoresis on a 0.8% Agarose/TBE gel. The gel was photographed under UV illumination and then DNA was transferred overnight in 0.4 M NaOH onto a positively charged nylon membrane (Nytran SuPerCharge, Whatman, Maidstone, UK). A P^32^-dCTP-labelled screening probe (5′Fgf22), corresponding to 400 bp of *fgf22* genomic sequence upstream of the 5′ homology arm, was prepared using Ready-To-Go DNA Labelling Beads (GE Healthcare, Amersham, UK) according to the manufacturer’s instructions. The membrane was hybridized with 50 ng of ^32^P-labelled probe, exposed overnight to a storage phosphor screen and read on a Storm 860 phosphorimage scanner (Molecular Dynamics, Sunnyvale, Ca, USA).

### Genotyping

Genomic DNA isolated from ear snips or tail biopsies was used for PCR genotyping. Primers for the wild type (5′-TTGTGAACCAAGATGGCAGG-3′ and 5′-CCAGCTTTGGACTTCATCTG-3′) and knockout (5′-TCGACTAGAGGATCAGCTTG-3′ and 5′ CAGGCCAGCATAGTCTACTT-3′) *fgf22* alleles generated 286 bp and 130 bp PCR products, respectively.

### RT-PCR

RT-PCR was performed to confirm FGF22 transcript deletion in *fgf22* knockout mice. Total RNA was isolated from brains of adult wild type, heterozygous and knockout animals using TRI reagent (Sigma, Poole, UK), and reverse transcribed (SuperScript™ II RT, Invitrogen, Paisley, UK) using random hexamer primers. RT-PCR was performed using primers (5′-ATTCTAGAATGCGCAGCCGCCTCTGG-3′ and 5′-ATGGGCCCTCAAGACGAGACCAAGAC-3′) that amplified a 482 bp fragment of the FGF22 cDNA. *GAPDH* was amplified as a control housekeeping gene using published primers for mouse and human sequence [19], [35].

### Realtime RT-PCR

Realtime RT-PCR was performed to assess relative mRNA expression levels of Fgf7 and Fgf10 in Fgf22 wild type and knockout mice. RNA extraction and reverse transcription was performed as above. Similarly, expression levels of FGF22 and NRF2 were quantified in HaCaT cells treated with scrambled control or FGF22 targeting siRNA pools. RNA was isolated using TRI reagent according to the manufacturer’s instructions and as for skin samples. Primers used were Fgf7 *(* 5′-GAACAAAAGTCAAGGAGCAACC-3′ and 5′-GTCATGGGCCTCCTCCTATT-3′), Fgf10 (5′-GAGAAGAACGGCAAGGTCAG-3′ and 5′-CTCTCCTGGGAGCTCCTTTT-3′) and NRF2 [30]. FGF22 primers were as for RT-PCR. All reactions were performed in triplicate. A negative control was performed with all the mix components except the cDNA sample. GAPDH primers were used as internal control. The reaction was performed using SYBR green (Qiagen, Crawley, UK) in a StepOne Plus Realtime PCR System (Applied Biosystems, Life technologies, USA) thermal cycler according to manufacturer’s instructions. The results were evaluated using the 2^−ΔΔct^ method [36].

### Histological Analysis

Normal and wounded back skin and tail skin were harvested and processed as described previously (Grose et al., 2007). Sections (4 µm) were analyzed using Haematoxylin/Eosin, anti-5′bromodeoxyuridine (BrdU) and Sirius red staining.

### Hair Analysis

Hair subtypes were analyzed by plucking hairs from the middle of the back of five eight weeks old wild type and *fgf22* knockout female mice (n = 100 hairs per genotype). They were measured and classified as guard, awl or auchene based on their appearance [26] under a light microscope (Axiophot, Zeiss, Welwyn, UK).

### Immunohistochemistry

For detection of proliferating cells labelled with BrdU, paraffin sections (4 µm) were incubated with a peroxidase-conjugated monoclonal antibody directed against BrdU (1∶500 dilution; Abcam, Cambridge, UK) for 1 h at room temperature. The signal was amplified using a StreptABComplex/HRP kit (Dako, Ely, UK) according to the manufacturer’s instructions, before peroxidase detection with a 3,3′- Diaminobenzidine substrate kit (Dako). Sections were counterstained with Mayer’s haemalum and mounted with Permount (Fisher Scientific, Loughborough, UK).

### BrdU Scoring

Cells exhibiting brown nuclear staining or showing mitotic figures were included in deriving the proliferation score. Cells stained in any of the categories 1 (faint), 2 (moderate) and 3 (strong) were considered positive whereas cells showing an absence of brown nuclear staining were considered negative. The percentage positive BrdU score was calculated by counting the total number of positive and negative cells over a specified field and then deriving the percentage of positive cells. A field was defined as the grid area provided in the microscope graticule (10×10 squares) under a x400 magnification (10x ocular and 40x objective). Ten fields per sample were counted. The fields to be included in the score were chosen on the basis of the observer’s interpretation of fields best representing the heterogeneity of wound tissue across the sample.

### Sirius Red Staining

To evaluate collagen organization in healing wounds, paraffin sections of full thickness 14 day wounds of *fgf22* wild type and knockout mice were stained with Sirius red. In bright-field microscopy, Sirius red stains collagen fibres in red on a pale yellow background. The same slides, when examined through a microscope with cross-polarised light show that the larger collagen fibres (collagen type I) are bright yellow or orange, and the thinner ones, including reticular fibres and collagen type III, are green. The more mature and well organized the collagen fibres, the greater the birefringence intensity. De-waxed and hydrated paraffin wound sections were stained in Picro-sirius red (0.1% Sirius Red F3B in saturated aqueous solution of Picric Acid) for one hour, then washed in two changes of acidified water (0.5% Glacial Acetic Acid), dehydrated and mounted as above.

### Tail Epidermis Wholemounts

Epidermal wholemounts were prepared by peeling tail skin off the bone, after making a scalpel incision along the length of the tail, and cutting into 5 mm^2^ pieces. These were incubated in 5 mM EDTA in PBS for 4 h at 37°C. Epidermal sheets were peeled away from the dermis and fixed in 1% Acetic Acid/95% overnight at 4°C. After rinsing in 100% ethanol, specimens were rehydrated through an ethanol series to water, transferred to PBS, counterstained for 10 seconds in a 1∶4 dilution of Mayer’s haemalum/water and mounted in 30% Glycerol in PBS.

### Primary Keratinocyte Culture

Primary keratinocytes were isolated from back skin of adult mice. Hair from the dorsal skin of a mouse, killed by cervical dislocation, was removed with electric clippers and depilatory cream (Nair, Church & Dwight, New Jersey, US), applied to the skin surface for approximately 3 minutes. Excess cream was removed and the skin was washed under running water. Next, mice were washed twice for 2 minutes in Iodine solution, rinsed in sterile water and washed for 2×2 minutes in 70% ethanol. Skin was then cut from neck to tail and peeled off using forceps. Skin was placed, epidermis side down, on a sterile surface and the hypodermis was carefully scraped off using a scalpel blade. Next, the skin was stretched flat and a piece of autoclaved no. 1 filter paper (Whatman) was applied onto the exposed surface of the dermis. The skin, including the attached paper, was cut into 10 mm wide strips using sterile scissors and floated, dermis side down, on the surface of 1% Trypsin in PBS, and incubated for 2 h at 37°C. The epidermis was peeled off using curved forceps and placed into ice-cold PCT Epidermal Keratinocyte Medium containing 1.3 mM Ca^2+^ (Millipore, Watford, UK). The epidermal sheets were dispersed in the medium using sterile scissors, until pieces were small enough to enter the tip of a 10 ml pipette. The resulting suspension was triturated by pipetting up and down 30 times, then transferred into a 50 ml conical tube on ice, leaving behind most of the cornified squames. The cell suspension was centrifuged at 200×g for 5 minutes. The cell pellet then was resuspended in high calcium medium (1.3 mM; CnT-07CF, Millipore) and filtered through a 100 µm cell strainer (BD Biosciences, Oxford, UK) into a new 50 ml conical tube. Cells were centrifuged at 200×g for 5 minutes and the cell pellet was resuspended in 10 ml high calcium medium. The cells were counted, centrifuged again and resuspended in the desired volume of low calcium medium (0.05 mM) to obtain a final cell concentration of 10^6^ cells/ml. Cells were plated on Collagen I coated 60 mm dishes (VWR, Lutterworth, UK), at a concentration of 10^6^ cells per dish.

Primary keratinocytes were incubated in 7% CO_2_ overnight at 36°C. The next day, the medium containing unattached cells was aspirated, the dish was rinsed with calcium/magnesium free PBS (Sigma) and fresh low calcium media was added. Thereafter cells were maintained in low calcium medium, with medium changed every two days.

### *In vitro* Scratch Wound Assay

Primary keratinocytes were seeded at a density of 10^6^ cells per well in Collagen I pre-coated 6-well plates (VWR) and grown to near confluency, when the culture medium was replaced with starving medium (1% FCS) for 2 hours. Scratch wounds were made, using a sterile 200 µl micropipette tip to form a cross in each well, and medium was replaced with low calcium (0.05 mM) medium (CnT-07CF, Millipore) containing 8% calcium free FCS (Biosera, Ringmer, UK). To determine the rate of cell migration, images were acquired at 0, 12, 18 and 32 h post-wounding using a Time-lapse microscope set up (Zeiss). Scratch widths were measured at 20 points along their length using ImageJ software version 1.429 (National Institute of Health, Wayne Rasband, USA). The percentage of migration was determined relative to the scratch width at 0 hours.

### *In vivo* Wound Healing

Full-thickness excisional wounds were generated in shaved dorsal skin of 6–8 weeks old mice using a 3 mm biopsy punch. Wounds were examined daily and collected after 0, 1, 5, 14, and 21 days for analysis. Mice were housed in isolator cages of up to 3 mice, control and knockout animals were housed separately. For each time point, 3 female and 3 male mice of each genotype were analysed. One hour prior to culling, mice were injected with BrdU (0.25 mg/kg; Sigma). Wounds were excised together with 2 mm of surrounding tissue and one wound per mouse was placed on a nitrocellulose membrane (GE Healthcare) and fixed overnight in 1% Glacial Acetic Acid/99% Ethanol at 4°C for histological analysis. Morphometric analysis of wound repair was performed on Haematoxylin/Eosin stained paraffin sections using ImageJ software. The wound gap was calculated as the distance between two margins of the inward growing epithelium and was measured three times for each wound section. The area of epithelial tongue also was determined using ImageJ 1.429 software (National Institute of Health, Wayne Rasband, USA) to outline the entire wound epithelium, starting from the most wound proximal hair follicle.

### Skin Carcinogenesis

Cohorts of seven weeks old female wild type and *fgf22* knockout mice were subjected to two-step skin carcinogenesis protocol performed as described previously [19]. Female mice (n = 10 per group) were treated, at eight weeks of age, with topically administered DMBA (25 µg in 200 µl acetone; Sigma), or vehicle alone as a control. From 9 weeks old, all mice were treated weekly with TPA (7.4 µg in 200 µl acetone; Sigma) for 15 weeks. Mice were monitored on a daily basis and papilloma/tumour counts were recorded weekly for up to 58 weeks post-initiation, at which stage the experiment was terminated. If mice appeared sick or the lesion reached a predefined limit of 14 mm diameter, they were killed and examined post-mortem. Skin lesions and macroscopically normal skin were harvested and fixed overnight in 10% Buffered Formalin for histological analysis. All animal experiments were approved by Cancer Research UK London Research Institute ethics committee and carried out under Home Office licence (70/5387, 70/5878), according to Institutional guidelines at Cancer Research UK London Research Institute.

### HaCaT Cells

HaCaT cells, an immortalized human keratinocyte line (kind gift of Dr P. Boukamp; [27]), were cultured in DMEM supplemented with 10% foetal bovine serum at 37°C, and 8% CO_2_. Cells were passaged weekly in T75 flasks and all the experiments were performed with sub-confluent cells. HaCaT cells at 70% confluence were stimulated with hFGF7, hFGF10 (PeproTech, London, UK) or hFGF22 (Cambridge Bioscience) at a concentration of 100 ng/ml, co-treated with Heparin (300 ng/ml). FGF dependent stimulation was blocked by 30 minute pre-treatment with the FGFR specific inhibitor, PD173074 (1.7 µM; Sigma).

### Western Blot Analysis

Cultured cells were lysed with NuPAGE LDS Sample buffer 2x (Invitrogen; supplemented with 100 mM DTT) for 5 minutes at room temperature, prior to scraping and brief sonication. Lysates were loaded in 4–12% gradient NuPAGE gels and run at 110 V at room temperature in NuPAGE MES running buffer (Invitrogen). Proteins were transferred onto nitrocellulose membranes (Schleicher & Schuell, Whatman) by electro-blotting for 3 h (4°C) at 30 V in Tris/Glycine buffer with 20% methanol and transfer was confirmed with Ponceau Red (Sigma) staining of the membranes. After de-staining in distilled water, membranes were incubated in blocking buffer (5% powdered milk in TBS) for 30 minutes at room temperature. Membranes were then incubated with 1° antibody overnight at 4°C. After rinsing in TBS containing 0.1% Tween-20 (3×5 minutes), membranes were incubated with 2° antibody for 1 h at room temperature, rinsed in TBS with 0.1% Tween-20 (3×5 minutes), and developed (ECL Chemiluminescence; GE Healthcare). Antibodies used were as follows; anti-phospho-p44/42 MAPK (Cat. #4370, Cell Signaling, New England Biolabs, Hitchin, UK) and anti-MAPK1 (Cat. #05-957, Upstate, Millipore). Primary antibodies were diluted 1∶1000 in TBS +3% BSA and incubate overnight at 4°C. Secondary antibodies (Dako), coupled with horseradish peroxidase, were used at a 1∶1000 dilution, incubated for 1 h at room temperature. Densitometric analysis was performed using ImageJ 1.429 software. Signal density was normalized to anti-ERK antibody as a loading control, for at least three separate treatments.

### RNA Interference

SiRNA used (Dharmacon, Cramlington, UK): Genome smart pool for human FGF22, M-013171. Cells (40–50% confluent in 6-well plates) were transfected for 4 h with siRNA (10 nM) using 4 µl Interferin (Polyplus transfection; PeqLab, Fareham UK), in a total reaction volume of 1.1 ml in each well of a 6-well plate. Messenger RNAs and functional activity were assayed 48 h post-transfection, and compared with mock and/or control siRNA-treated cells (SiControl Non-Targeting siRNA pool; D-001210 Dharmacon).

## Supporting Information

Figure S1 **RNAi-mediated knock down of FGF22 in HaCaT cells.** HaCaT cells were subject to mock transfection (Ctr) or transfection with 10 nM control non-targeting siRNA (Scr) or siRNA to FGF22 (1 nM or 10 nM). Messenger RNA levels for FGF22 and NRF2 were measured by Realtime PCR, normalised to GAPDH. Samples were run in triplicate and a representitive repeat is shown. FGF22 RNA levels were reduced by 50% following RNA interference, but levels of NRF2 expression were unaffected.
(TIF)

Figure S2 **Expression of Fgf7 and Fgf10 mRNA in** ***fgf22*** **wild type and knockout mice.** Realtime PCR on RNA samples isolated from back skin of eight-week-old mice showed no significant difference in the expression levels of Fgf7 or Fgf10 mRNA between control mice (Ctr) and null mice (−/−). Results were normalised to GAPDH as a control for RNA concentration and integrity. Experiments were carried out on 6 female mice and error bars represent standard error among the samples.
(TIF)
