# Supplementary figures and images for: Fibroblast Growth Factor 22 Is Not Essential for Skin Development and Repair but Plays a Role in Tumorigenesis
Source: PLoS One. 2012 Jun 21;7(6):e39436. doi: 10.1371/journal.pone.0039436 (PMC3380851; doi:10.1371/journal.pone.0039436)

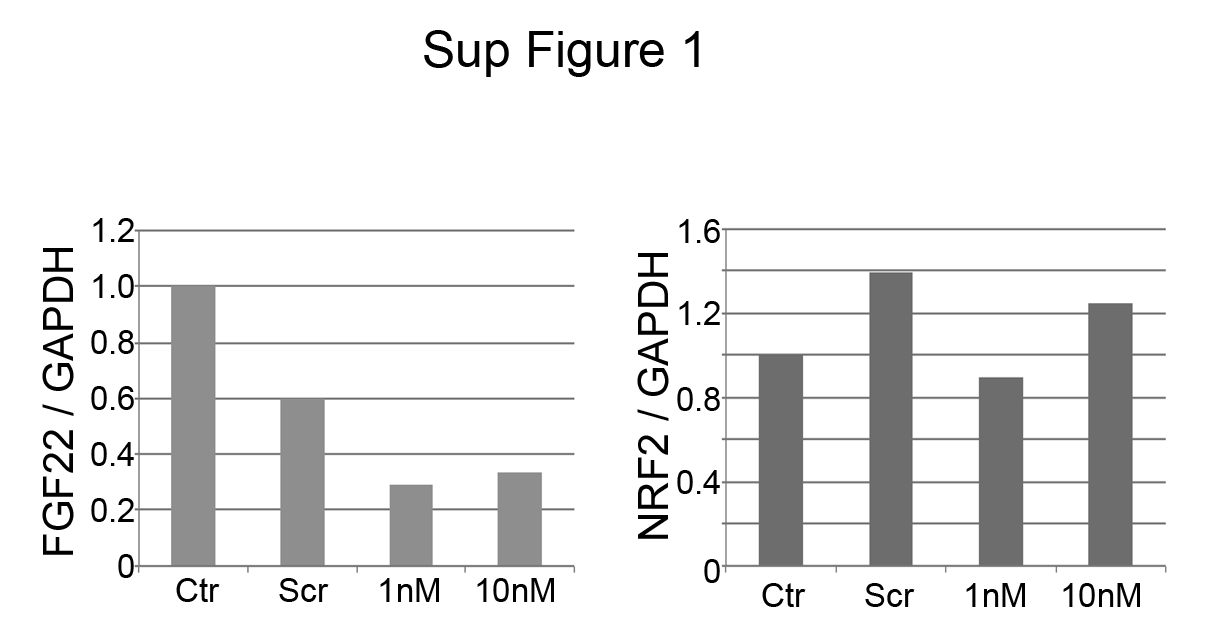

Supplement: Figure S1 — RNAi-mediated knock down of FGF22 in HaCaT cells. HaCaT cells were subject to mock transfection (Ctr) or transfection with 10 nM control non-targeting siRNA (Scr) or siRNA to FGF22 (1 nM or 10 nM). Messenger RNA levels for FGF22 and NRF2 were measured by Realtime PCR, normalised to GAPDH. Samples were run in triplicate and a representitive repeat is shown. FGF22 RNA levels were reduced by 50% following RNA interference, but levels of NRF2 expression were unaffected. (TIF) [file pone.0039436.s001.tif]

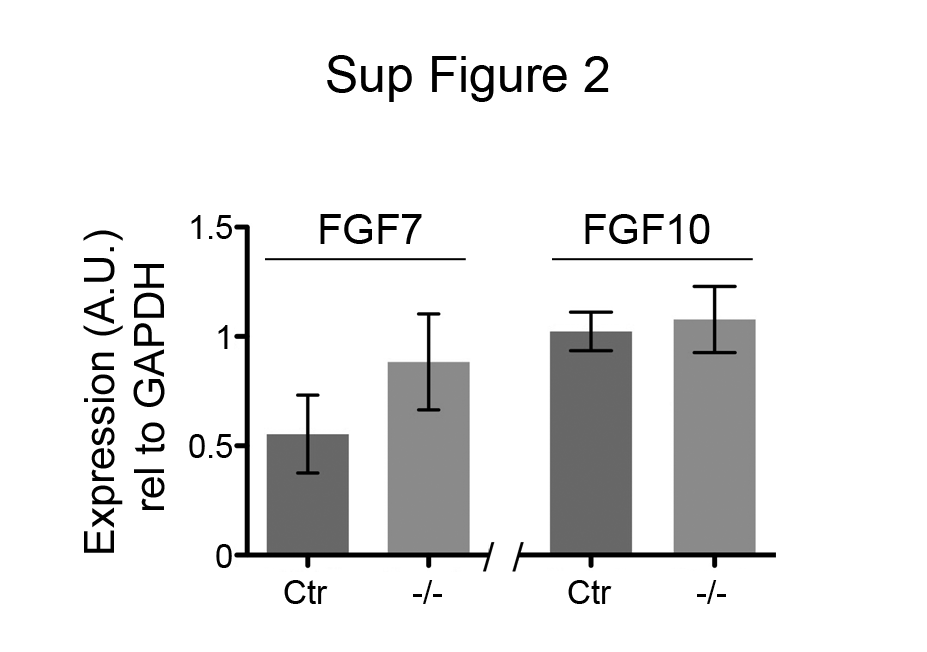

Supplement: Figure S2 — Expression of Fgf7 and Fgf10 mRNA in fgf22 wild type and knockout mice. Realtime PCR on RNA samples isolated from back skin of eight-week-old mice showed no significant difference in the expression levels of Fgf7 or Fgf10 mRNA between control mice (Ctr) and null mice (−/−). Results were normalised to GAPDH as a control for RNA concentration and integrity. Experiments were carried out on 6 female mice and error bars represent standard error among the samples. (TIF) [file pone.0039436.s002.tif]
